# Extracellular Nicotinamide Phosphoribosyltransferase as a Surrogate Marker of Prominent Malignant Potential in Colonic Polyps: A 2-Year Prospective Study

**DOI:** 10.3390/cancers15061702

**Published:** 2023-03-10

**Authors:** Tsung-Hsing Chen, Hung-Chih Hsu, Jeng-Fu You, Cheng-Chou Lai, Yung-Kuan Tsou, Chia-Lin Hsu, Cathy S. J. Fann, Rong-Nan Chien, Ming-Ling Chang

**Affiliations:** 1 Division of Hepatology, Department of Gastroenterology and Hepatology, Chang Gung Memorial Hospital, Taoyuan 33305, Taiwan; 2 Department of Medicine, College of Medicine, Chang Gung University, Taoyuan 33305, Taiwan; 3 Division of Hematology-Oncology, Chang Gung Memorial Hospital, Taoyuan 33305, Taiwan; 4 Colorectal Section, Department of Surgery, Chang Gung Memorial Hospital, Taoyuan 33305, Taiwan; 5 Institute of Biomedical Sciences, Academia Sinica, Taipei 115, Taiwan

**Keywords:** visfatin, PBEF, advanced colonic polyp, colonoscopy

## Abstract

**Simple Summary:**

The implications of extracellular nicotinamide phosphoribosyltransferase (eNAMPT) in colonic polyps remain uncertain. A 2-year prospective cohort study was conducted. Of 532 patients, 80 (15%) had prominent malignant potential (PMP) in colonic polyps, including villous adenomas, adenomas with high-grade dysplasia, and adenocarcinomas. Baseline associations were observed: colonic polyp pathology, total cholesterol, and neutrophil-to-lymphocyte ratio with eNAMPT levels, and age, polyp size, and eNAMPT levels with polyp pathology. Baseline eNAMPT levels were higher in patients harboring polyps with PMP than in patients without PMP, and baseline eNAMPT levels predicted PMP (cutoff: >4.238 ng/mL). Proportions of eNAMPT-positive glandular and stromal cells were higher in polyps with PMP than in polyps without PMP. eNAMPT levels decreased within 48 weeks postpolypectomy and remained stable afterward regardless of PMP until 96 weeks postpolypectomy. However, those with PMP had a higher degree of eNAMPT decline within 24 weeks. With a link to inflammation and lipid metabolism, along with its decreasing trend after polypectomy, serum eNAMPT may serve as a surrogate marker of PMP in colonic polyps. In situ probing of the NAMPT-associated pathway holds promise in attenuating PMP, as much of the eNAMPT likely originates from colonic polyps.

**Abstract:**

Background/aims: The implications of extracellular nicotinamide phosphoribosyltransferase (eNAMPT), a cancer metabokine, in colonic polyps remain uncertain. Methods: A 2-year prospective cohort study of patients who underwent colonoscopy was conducted. Biochemical parameters and serum eNAMPT levels were analyzed at baseline and every 24 weeks postpolypectomy. NAMPT-associated single-nucleotide polymorphisms (SNPs), including rs61330082, rs2302559, rs10953502, and rs23058539, were assayed. Results: Of 532 patients, 80 (15%) had prominent malignant potential (PMP) in colonic polyps, including villous adenomas (n = 18, 3.3%), adenomas with high-grade dysplasia (n = 33, 6.2%), and adenocarcinomas (n = 29, 5.5%). Baseline associations were as follows: colonic polyp pathology (*p* < 0.001), total cholesterol (*p* = 0.019), and neutrophil-to-lymphocyte ratio (*p* = 0.023) with eNAMPT levels; and age (*p* < 0.001), polyp size (*p* < 0.001), and eNAMPT levels (*p* < 0.001) with polyp pathology. Higher baseline eNAMPT levels were noted in patients harboring polyps with PMP than in patients without PMP (*p* < 0.001), and baseline eNAMPT levels significantly predicted PMP (cutoff: >4.238 ng/mL, *p* < 0.001). Proportions of eNAMPT-positive glandular and stromal cells were higher in polyps with PMP than in polyps without PMP (64.55 ± 11.94 vs. 14.82 ± 11.45%, *p* = 0.025). eNAMPT levels decreased within 48 weeks postpolypectomy (*p* = 0.01) and remained stable afterward regardless of PMP until 96 weeks postpolypectomy. However, those with PMP had a higher degree of eNAMPT decline within 24 weeks (*p* = 0.046). All investigated SNPs were in linkage disequilibrium with each other but were not associated with eNAMPT levels. Conclusion: With a link to inflammation and lipid metabolism, along with its decreasing trend after polypectomy, serum eNAMPT may serve as a surrogate marker of PMP in colonic polyps. In situ probing of the NAMPT-associated pathway holds promise in attenuating PMP, as much of the eNAMPT likely originates from colonic polyps.

## 1. Introduction

Colorectal cancer (CRC) is the third most common cancer in men and the second most common cancer in women worldwide [1]. Most CRCs arise from colonic polyps, especially adenomatous polyps [2]. Polyp number, size, and pathological findings are crucial for CRC. Advanced adenomas are defined as those that are >1 cm or those that contain appreciable villous tissue or high-grade dysplasia [3,4]. The efforts to control CRC focus mainly on strategies to reliably detect and resect advanced adenomas before they become malignant [4]. Currently, colonoscopy is the gold standard for detecting colonic polyps and affords an opportunity for therapy through polypectomy and histological diagnosis. Nevertheless, colonoscopy may not detect all colonic polyps, particularly small polyps and those located in the proximal colon [5]. Missed lesions potentially lead to more than half of all interval cancers diagnosed 3–5 years after the index procedure [6]. Furthermore, in 5%–10% of patients, usually those with diverticulosis or previous pelvic surgery, the endoscopist may not be able to pass the instrument comfortably and safely to the cecum [7]. Although some epigenetic, proteomic, and fecal DNA markers for the early detection of CRC and polyps have been reported [8,9], a noninvasive marker of advanced polyps that aids in decreasing the misdiagnosis rate of colonoscopy is currently unavailable.

Nicotinamide phosphoribosyltransferase (NAMPT) is a regulator of the intracellular nicotinamide adenine dinucleotide (NAD) pool. Through its NAD biosynthetic activity, NAMPT influences the activity of NAD-dependent enzymes [10], thereby acting as a driver or pacemaker that maintains the pace of metabolism throughout the body [11]. NAMPT enhances cellular proliferation and tips the balance toward cell survival following a genotoxic insult; moreover, NAMPT is considered a molecular link among metabolism, inflammation, and cancer [12]. NAMPT is found both extracellularly (eNAMPT) and intracellularly (iNAMPT). Specifically, eNAMPT is a highly conserved 52-kDa protein and is responsible for transmitting interorgan signals [13]. iNAMPT is associated with cell aging and cell survival [14]. In addition to its enzymatic function, eNAMPT exhibits cytokine-like activity and is considered an adipokine. Thus, eNAMPT is also called pre-B-cell colony-enhancing factor or visfatin [15]. Both the liver and peripheral blood leukocytes contain maximal eNAMPT mRNA transcript levels, but the eNAMPT protein is ubiquitously expressed in all tissues [16]. eNAMPT regulates glucose-stimulated insulin secretion in pancreatic β cells through NAD intermediates [17]. However, it does not appear to control glucose metabolism in some conditions but may play a regulatory role in lipid metabolism [18]. For example, a positive correlation between eNAMPT and total cholesterol (TC) levels was shown in postmenopausal women [19], women with polycystic ovary syndrome [20], and hepatitis C virus-infected patients [21]. In nondiabetic adults, NAMPT mRNA is a proinflammatory marker of adipose tissue associated with systemic insulin resistance and hyperlipidemia [22]. Altogether, eNAMPT has endocrine, paracrine, and autocrine actions [23]. Interestingly, fat-mediated alterations of the gut microbiota link bile acid metabolism to CRC risk [24]. Bile acids are involved in the absorption of fat and the regulation of lipid homeostasis [25], and chronic inflammation, such as inflammatory bowel disease, exposes these patients to a number of signals known to have tumorigenic effects [26]. Namely, similar to eNAMPT, CRC risk is also highly associated with lipid metabolism and inflammation. To date, associations between eNAMPT and oncogenic potential have been uncovered in several cancers, including CRC [27], esophagogastric junction adenocarcinoma [28], melanoma [29], chronic lymphocytic leukemia [30], myeloproliferative neoplasm-associated myelofibrosis [31], rhabdomyosarcomas, and leiomyosarcomas [32]. Therefore, eNAMPT is defined as a new cancer metabokine. However, the implications of eNAMPT in colonic polyps remain uninvestigated. Thus, we sought to elucidate these implications by conducting a prospective study to analyze eNAMPT profiles by adjusting for crucial confounders in patients with colonic polyps who underwent polypectomy with an available pathological diagnosis.

## 2. Materials and Methods

### 2.1. Patients

The study group comprised patients who were > 18 years old and underwent screening, surveillance, or therapeutic colonoscopy. Specifically, the patients were referred from our health checkup center for a first-time polypectomy, had a first-degree relative with a history of CRC, and had a positive fecal test, abdominal pain, and unexplained weight loss.

Subjects with a history of bowel resection and inflammatory bowel diseases and suspicion of polyposis syndrome; those with infections with human immunodeficiency virus, hepatitis C virus, or hepatitis B virus; those with hemochromatosis, acromegaly, or cancers, or who were recipients of solid organ transplants; those on immunomodulatory, lipid-lowering, glucose-lowering, or anti-hypertensive drugs; and those who had undergone incomplete colonoscopy (failure to intubate the colon/cecum) and poor bowel preparation, as assessed with the Aronchick scale [33], were excluded.

Colonoscopy was performed by experienced endoscopists using an electronic endoscopic reporting system.

### 2.2. Study Design

A total of 532 patients were recruited at a tertiary referral center between December 2014 and December 2020. All patients were naive patients who had not previously undergone polypectomy or colon surgery (63 underwent a screening colonoscopy, 50 patients received a surveillance colonoscopy due to a positive fecal occult blood test, and the others (n = 419) underwent a therapeutic colonoscopy following transfer from the check-up center after a screening colonoscopy). The patients underwent bowel preparation with a split-dose regimen of either 2 L of polyethylene glycol electrolyte solution or 300 mL of sodium picosulfate/magnesium citrate preparation–Bowklean solution [34]. During the colonoscopy, sedation was induced by the administration of intravenous midazolam and fentanyl at the endoscopist’s discretion. Before colonoscopy, some factors, including body mass index (BMI), fasting triglycerides (TGs), total cholesterol (TC), homeostasis model assessment-estimated insulin resistance (HOMA-IR) [fasting insulin (μU/mL) × fasting glucose (mmol/l)/22.5], high-density lipoprotein cholesterol (HDL-C), uric acid (UA), carcinoembryonic antigen (CEA), high-sensitivity C-reactive protein (HS-CRP), alanine aminotransferase (ALT), neutrophil-to-lymphocyte ratio (NLR), estimated glomerular filtration rate (eGFR), and eNAMPT levels (R&D Systems, Minneapolis, MN, USA), were measured for all patients. After polypectomy, all the abovementioned measurements were performed every 24 weeks during the follow-ups. The genotypes of NAMPT-associated SNPs, including rs61330082 [35], rs2302559 [36], rs2058539, and rs10953502 [37], were assessed as described previously [38] (Supplementary Table S1) or were assessed using TaqMan SNP Genotyping assays (Applied Biosystems, Waltham, MA, USA) ( Supplementary Table S2). Serum biochemical measurements were conducted in research laboratories or the clinical pathology department of the hospital using routine automated techniques. All detected polyps were removed and sent in individual jars for pathological assessment. Polyp location (left colon: descending and sigmoid colons and rectum; right colon: transverse and ascending colons and cecum), size, and number were recorded. The polyp sizes were estimated using a closed jumbo biopsy forceps tip (2.4 mm) or snare catheter (2 mm). In the case of multiple polyps, only the records of the largest polyp were analyzed (categories used for classification of size, histopathology, and location).

### 2.3. Polypectomy or Surgical Removal of Polyps

Polyps were removed by surgery, endoscopic mucosal resection, endoscopic submucosal dissection, biopsy forceps, or polypectomy, as indicated.

### 2.4. Immunohistochemical (IHC) Studies of Colonic Polyps

IHC studies of colonic polyps were performed in paraffinized samples. The pathological findings were coded according to the degree of their malignant potential as follows, with codes 0–2 indicating negligible malignant potential [4]: 0, no polyp; 1, inflammation, muscle prolapse, or juvenile polyp; 2, hyperplastic polyp; 3, sessile serrated adenoma (SSA); 4, tubular adenoma; 5, tubulovillous adenoma; 6, villous adenoma; 7, adenoma with high-grade dysplasia; and 8, adenocarcinoma. The randomly selected cases of each pathological entity (0–8) were stained with eNAMPT using antibodies against eNAMPT (Lifespan BioScience Inc., Seattle, WA, USA) according to the manufacturer’s protocol. All polyp pathology was reviewed by gastrointestinal pathologists at participating sites who were blinded to study participation and polyp prediction details. In addition, IHC for eNAMPT was performed using peripheral blood (PB) smears according to the manufacturer’s protocols [29]. After serial centrifugation of PB, smears of isolated peripheral leukocytes were prepared. The intensity of eNAMPT expression in IHC was assessed using ImageJ software (http://imagej.nih.gov/ij/ (accessed on 1 January 2022), National Institutes of Health, USA) for samples from 20 patients of each polyp code (0–8).

### 2.5. Statistical Analysis

All statistical analyses were performed using either MedCalc (MedCalc ver. 12.4, MedCalc Software Corp., New York, NY, USA), Statistical Analysis System (SAS version 9.4), PLINK (version 1.07), HAPLOVIEW (version 4.2), Statistical Package for the Social Sciences (SPSS ver. 21.0, SPSS Inc., Chicago, IL, USA), or MassARRAY Typer 4.0 (Sequenom) software. By adjusting for all independent variables with a *p* value < 0.1 in the univariate analyses, multivariate linear regression models were used to assess the relationship between various dependent and independent factors. When comparing variables from multiple groups, data were analyzed by one-way analysis of variance (ANOVA). Post hoc analysis was performed with least significant difference multiple comparison analysis. Variables measured before and at every 24 weeks after polypectomy were analyzed and compared with ANOVA employing general linear model-repeated measures. The performance of eNAMPT in predicting the malignant potential of colonic polyps was analyzed by calculating the area under the receiver-operating characteristic (ROC) curve (AUC) and compared using Delong’s test. The sensitivities and specificities were compared using McNemar’s paired comparison test. For the genetic analyses, SNPs of poor quality were removed using a sequentially exclusive procedure. First, samples with a genotyping call rate <0.9 were removed. Next, SNPs with a minor allele frequency (MAF) < 0.01 were removed. Finally, SNPs with an adjusted *p* value < 0.05 that controlled for the false discovery rate [39] in an exact Hardy–Weinberg equilibrium test [40] were removed. Genotype association tests were performed as described previously [41]. The associations between the tested variables and the haplotype block were assessed using stepwise regression models.

### 2.6. Informed Consent

Written informed consent was obtained from each patient. The study protocol conformed to the ethical guidelines of the 1975 Declaration of Helsinki and was approved by the hospital institutional review board.

## 3. Results

### 3.1. Baseline Characteristics of Enrolled Patients

The baseline characteristics of the enrolled patients are shown in Table 1. Of the 532 patients, 379 (71.2%) were males and 153 (28.8%) were females, and the median and mean ages were 57.00 and 55.75 years, respectively. The median polyp number and size were 2 and 1.2 cm, respectively. In total, 80 (15%) polyps had prominent malignant potential (PMP) in their colonic polyps, including villous adenomas (n = 18, 3.3%), adenomas with high-grade dysplasia (n = 33, 6.2%), and adenocarcinomas (n = 29, 5.5%). The left colon contained 61.4% of the polyps. Most polyps <1 cm (n = 140) were tubular adenomas (67.7%) or hyperplastic polyps (19.5%), while 0.7% were villous adenomas (n = 1), 4.9% were adenomas with high-grade dysplasia (n = 7), and 0.7% (n = 1) were adenocarcinoma. Among the polyps >1 cm (n = 392), 53.6% were tubular adenomas, 17.8% were tubulovillous adenomas, 4.4% were villous adenomas (n = 17), 6.6% (n = 26) were adenomas with high-grade dysplasia, and 7.1% (n = 28) were adenocarcinomas.

### 3.2. Baseline Associations of Various Variables

**Figure 1:**
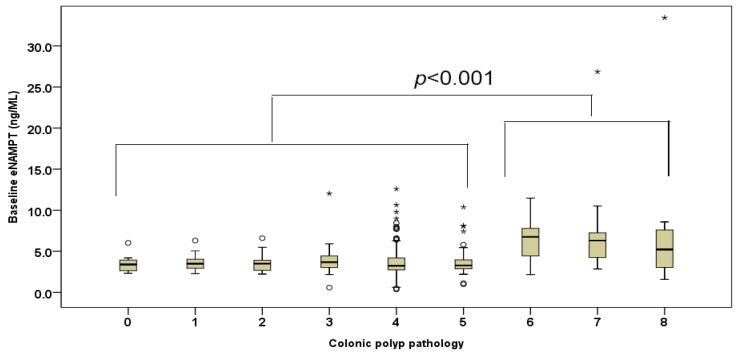
Bart charts (95% CI) of serum eNAMPT levels in patients stratified by polyp pathology. 0, no polyp; 1, inflammation, muscle prolapse, or juvenile polyp; 2, hyperplastic polyp; 3, sessile serrated adenoma (SSA); 4, tubular adenoma; 5, tubulovillous adenoma; 6, villous adenoma; 7, adenoma with high-grade dysplasia; and 8, adenocarcinoma.

**Figure 2:**
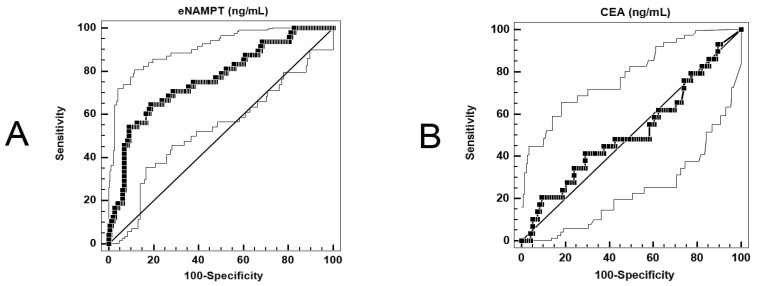
ROC curves for baseline eNAMPT (**A**) and CEA (**B**) in determining the malignant potential of polyps.

The multivariate analyses showed that the levels of baseline TC and NLR and pathology of the largest colonic polyp were independently associated with baseline eNAMPT levels (Table 2). Thus, we stratified baseline eNAMPT levels by the pathological findings of the colonic polyps. Patients with villous adenomas (group 6), adenomas with high-grade dysplasia (group 7) and adenocarcinomas (group 8) had higher eNAMPT levels than patients in the other groups (groups 1–5), while no differences in eNAMPT levels were found among patients in groups 6–8. Similarly, no differences could be identified between polyps in groups 1–5 (Figure 1). Thus, we reclassified the patients by the presence of PMP (groups 6–8) of colonic polyps and examined the efficacy of eNAMPT in predicting the PMP of colonic polyps. The AUC for eNAMPT in predicting the PMP of colonic polyps was 0.766 [95% confidence interval (CI): 0.716–0.810, sensitivity: 72.34%; specificity: 78.93%, *p* < 0.001], with a cutoff value of >4.238 ng/mL (Figure 2A). In contrast, the AUC for CEA in predicting the PMP of colonic polyps was only 0.534 (*p* = 0.5778), with a cutoff value of >2.22 ng/mL (Figure 2B).

The associations among the independent factors of baseline eNAMPT, including colonic pathology, TC and NLR, were as follows: baseline age, eNAMPT levels, and polyp size were associated with colonic polyp pathology ( Supplementary Table S3); baseline eNAMPT and HDL-C levels, platelet count, and polyp size and number were associated with baseline TC levels ( Supplementary Table S4); and baseline eNAMPT and HS-CRP levels were associated with baseline NLR ( Supplementary Table S5).

### 3.3. Genetic Study

All four investigated NAMP-associated SNPs were in high linkage disequilibrium (LD) with each other. Moreover, rs2302559, rs10953502, and rs23058539 were in the same LD block ( Supplementary Figure S1). However, none of them were associated with baseline eNAMPT or any other metabolic profiles, including TC and HOMA-IR levels (Supplementary Tables S3–S5).

### 3.4. Immunohistochemistry (IHC) of eNAMPT in Colonic Polyps and PB Smears

**Figure 3:**
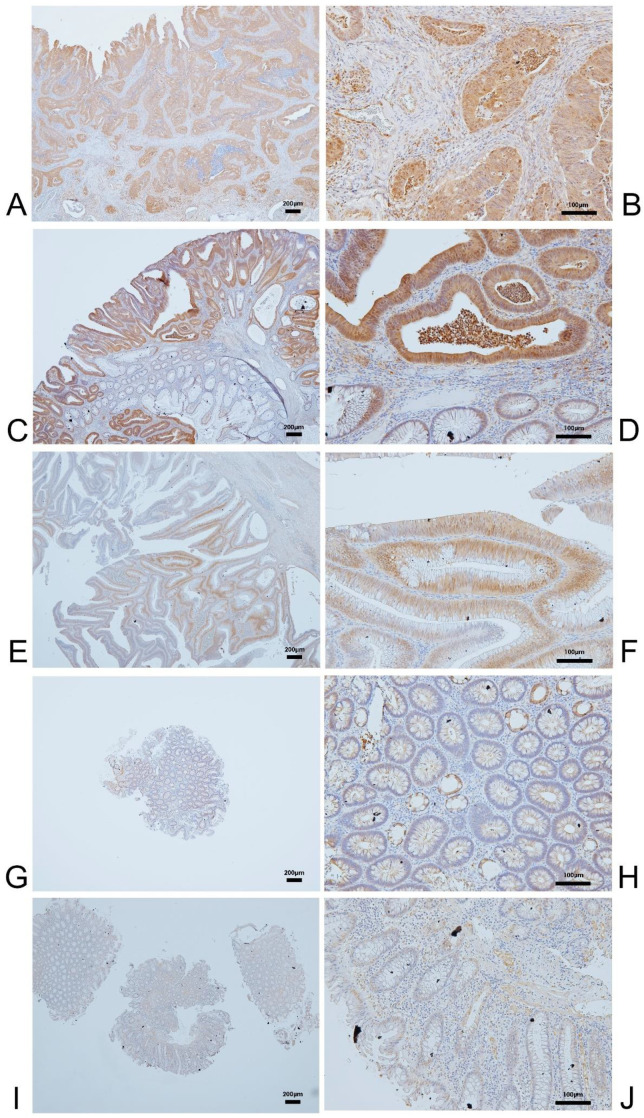
IHC of eNAMPT in colonic polyps. Representative cases of adenocarcinoma (**A**,**B**), adenoma with high-grade dysplasia (**C**,**D**), adenoma with villous components (**E**,**F**), simple adenoma (**G**,**H**), and hyperplastic polyps (**I**,**J**) are shown. (**A**,**C**,**E**,**G**,**I**) 40×; (**B**,**D**,**F**,**H**,**J**) 200×. The eNAMPT-positive cells are stained brown.

We compared the proportions of eNAMPT-positive cells between polyps with and without PMP. The quantitative proportions of eNAMPT-positive cells were higher in polyps with PMP than in those without PMP (64.55 ± 11.94% vs. 14.82 ± 11.45%, *p* = 0.025). Specifically, eNAMPT-positive cells were mainly detected in the lumen site. Some stromal cells, including inflammatory and endothelial cells, also expressed eNAMPT. Additionally, some cell clumps in glandular crypts expressed eNAMPT, especially in adenocarcinomas. For polyps with PMP, both the nuclei and cytoplasm of glandular cells expressed eNAMPT, whereas for simple adenomas or hyperplastic polyps, the nuclei of glandular cells expressed the majority of eNAMPT (Figure 3). However, peripheral blood (PB) smears showed similar ratios (48 ± 16 vs. 56 ± 19%, *p* = 0.368) of eNAMPT-positive leukocytes between the patients harboring polyps with and without PMP.

### 3.5. Alterations in eNAMPT Levels after Polypectomy

**Figure 4:**
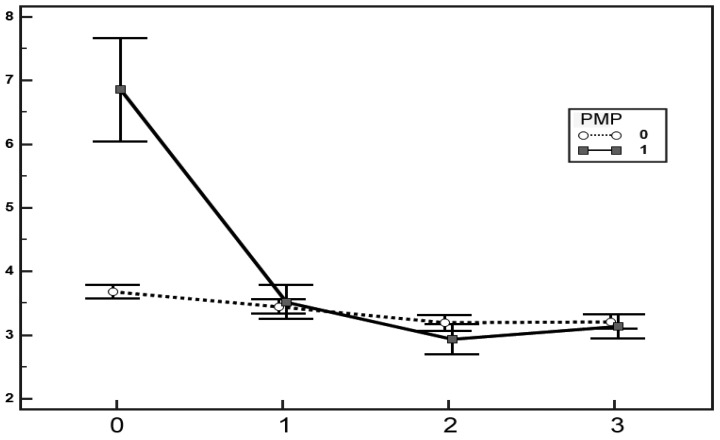
Longitudinal trends of the mean ± standard error for eNAMPT levels (ng/mL). Solid line: eNAMPT levels in patients with polyps with prominent malignant potential (PMP); dashed line: eNAMPT levels in patients with polyps lacking PMP. Time points: 0: baseline; 1: 24 weeks postpolypectomy; 2: 48 weeks postpolypectomy; 3: 96 weeks postpolypectomy.

During the follow-up period of up to 3 years (median: 2.61 years; mean: 2.57 years), compared with baseline levels, the eNAMPT levels decreased further within 48 weeks postpolypectomy and remained stable afterward, regardless of PMP. However, the degree of decline in eNAMPT levels was higher in patients with PMP than in those without PMP within 24 weeks (decline difference, *p* = 0.046). The former had higher baseline eNAMPT levels (6.19 ± 5.43 vs. 3.88 ± 1.97 ng/mL, *p* < 0.001), and the differences in eNAMPT levels between patients with and without PMP vanished at 24 weeks (*p* = 0.797), at 48 weeks (*p* = 0.103), and at 96 weeks postpolypectomy (Figure 4).

## 4. Discussion

Since most patients in the current cohort were transferred for therapeutic colonoscopy to undergo polypectomy, the polyp detection rate was as high as 97%, which is in contrast to the reported colonic polyp prevalence of 30% [4]. The findings that males accounted for the majority of colonic polyp cases and that the mean age was 55.75 years were consistent with the Asia Pacific consensus that male sex is a risk factor for colonic polyps and that screening for CRC should start at the age of 50 years [42]. Moreover, over half of the colonic polyps were located on the left side, which is in accordance with the literature [43], and the PMP rate of 15% was within the reported range [4].

To the best of our knowledge, this prospective study is the first to describe the implications of eNAMPT in colonic polyps. In addition to the established risk factors for CRC, including age [44] and polyp size [45], baseline eNAMPT levels were independently associated with polyp pathology. NLR is an inflammatory factor that indicates the prognosis of many cancers [46], and we coded the pathology of colonic polyps according to the degree of their malignant potential [4]. Thus, the associations of NLR and polyp pathology with eNAMPT levels are consistent with the role of eNAMPT as a link between inflammation and cancer [12]. In particular, the finding that baseline serum eNAMPT levels were associated with the PMP of colonic polyps is novel. Accordingly, the levels of eNAMPT in patients with CRC are higher than those in control individuals [47], especially those with advanced disease stages [27]. Although baseline CEA levels are reported to be risk factors for advanced colonic polyps [48,49], we did not find any role for CEA in highlighting the PMP of colonic polyps. In contrast, baseline serum eNAMPT levels >4.238 ng/mL indicated PMP, independent of polyp size. Interestingly, although most polyps <1 cm were not advanced polyps, 6.3% exhibited PMP. In contrast, among the polyps >1 cm, over 53.6% were simple adenomas. In clinical practice, polypectomy may be contraindicated in some patients due to risks of hemorrhage, perforation, or cardiovascular events [50]. In contrast to biopsy, which may miss residual polyps with undetected PMP, baseline eNAMPT levels are particularly valuable as a surrogate marker for small polyps with PMP or large polyps without PMP. Thus, eNAMPT levels may be used as a reference to determine the absolute indication for colonoscopy and polypectomy. There was no definite association between eNAMPT levels and HOMA-IR; instead, baseline TC levels were associated with eNAMPT levels. Collectively, in the presence of colonic polyps, the proinflammatory and lipid-regulatory role of eNAMPT is highlighted by its association with NLR and TC. All four of the investigated NAMPT-SNPs were in high LD with each other. However, none of them were associated with eNAMPT, TC, or HOMA-IR levels. The impact of these SNPs on eNAMPT expression may be negligible on the investigated variables in patients with colonic polyps. In a murine model, eNAMPT is partially released by melanoma [29], and the expression of eNAMPT is stronger in most primary human CRC tissue samples than in their normal counterparts [27]. Our IHC studies also demonstrated that eNAMPT-positive colonic polyp cells, especially those with malignant or premalignant characteristics, were the potential source of elevated eNAMPT levels in patients harboring polyps with PMP. This is consistent with the importance of NAMPT in supporting cancer cell metabolism, DNA repair, and proliferation [30,31], and in shaping the tumor microenvironment [27]. Furthermore, consistent with the role of eNAMPT as a proangiogenic molecule [51], some eNAMPT-positive stromal cells were endothelial cells. Both the cell cytoplasm and the nuclei of polyps with PMP prominently expressed eNAMPT, whereas in polyps without PMP, the cell nuclei primarily expressed eNAMPT. These findings indicate that eNAMPT was more readily secreted from polyps with PMP, which led to higher serum levels of eNAMPT. Furthermore, PB was less likely to be the source of high eNAMPT levels in patients with colonic polyps with PMP, as patients harboring polyps with and without PMP exhibited similar PB eNAMPT expression. The immunometabolic effects of eNAMPT may occur through in situ transcriptional or posttranscriptional regulation with the paracrine and autocrine [23] effects of colonic polyp cells. These results suggest the potential of targeting the in situ eNAMPT-associated pathway (probably via colonic mucosa) to reverse or prevent PMP in colonic polyps. Moreover, given that NAMPT is important for overall immune and metabolic regulation [20], locally targeting eNAMPT may be a feasible alternative to systemically blocking NAMPT, effectively avoiding perturbation of overall physiological homeostasis.

Longitudinally, eNAMPT levels decreased within 48 weeks postpolypectomy, regardless of PMP. Since the NLR was associated with the eNAMPT level, these universal decreases in eNAMPT levels likely highlight the abolishment of inflammation after the removal of polyps [52]. Additionally, the much higher degree of decline in eNAMPT levels 24 weeks postpolypectomy in patients with PMP than in those without PMP in polyps suggested the synergistic effects of PMP and inflammation in augmenting eNAMPT levels. This finding further supports the causal relationship between eNAMPT levels and the PMP of colonic polyps in a dynamic manner. In addition to polypectomy or operation, an increasing number of studies have supported the effectiveness of medical therapeutics, including Chinese herbal medicines (CHMs) [53,54], chemotherapy, immunotherapy, and targeted drugs [55] for the prevention or treatment of CRC. The dynamic changes in eNAMPT levels according to the PMP of colonic polyps indicate that eNAMPT potentially serves as a noninvasive tool to monitor the efficacies of the above medical treatments for CRC.

This study has limitations. First, the AUC for eNAMPT in predicting the PMP of colonic polyps was 0.766, which suggests acceptable but not excellent discrimination [56]. In addition to eNAMPT levels, other factors, such as age and polyp size, were also associated with the pathology of colonic polyps and might account for it. Second, due to the medical care-seeking patient-only nature, selection bias, particularly Berkson’s bias [57], is an inherent problem in the present study, which was based on a single medical center. Third, some risk factors for colon polyps, such as diet and exercise, cannot be assessed comprehensively in the current study, which is a limitation. A future prospective study with a large case number investigating crucial factors comprehensively from other institutes is needed to verify the role of eNAMPT for colonic polyp PMP.

## 5. Conclusions

In conclusion, since serum eNAMPT is a link to inflammation and lipid metabolism and its expression after polypectomy shows a decreasing trend, it may serve as a surrogate marker for the PMP of colonic polyps, independent of polyp size, number, and location. Since some eNAMPT may originate from colonic polyps, probing the in situ eNAMPT-associated pathway holds promise for preventing the development of colonic polyps with PMP.

## Figures and Tables

**Table 1:** Baseline characteristics of patients who underwent colonoscopy (median/mean ± standard deviation (range)).

Baseline Variables	Total (n = 532)
Male sex, n (%)	379 (71.2)
Female sex, n (%)	153 (28.8)
Age (yr)	57.00/55.75 ± 10.53 (24–85)
BMI	25.33/25.37 ± 3.55 (15.6–35.1)
HOMA-IR	1.19/1.55 ± 1.57 (0.16–13.87)
TC (mg/dL)	193.0/194.0 ± 35.14 (104–314)
TGs (mg/dL)	116.0/133.2 ± 75.2 (25–630)
HDL-C (mg/dL)	45.00/46.58 ± 11.36 (22–85)
TG/HDL-C	2.695/3.211 ± 2.279 (0.42–14.00)
UA (mg/dL)	6.00/6.010 ± 1.499 (2.8–11.0)
NLR	1.81/2.053 ± 1.349 (0.44–18.2)
HS-CRP (mg/dL)	1.340/2.925 ± 7.640 (0.2–109.91)
CEA (ng/mL)	1.24/1.85 ± 4.49 (1–65)
Platelet count (10^3^/µL)	227.0/229.4 ± 59.97 (41.00–498.0)
eNAMPT (ng/mL)	3.39/4.14 ± 2.86 (0.35–33.43)
ALT (U/L)	27.00/32.37 ± 24.78 (7–196)
eGFR (mL/min/1.73 m^2^)	87.93/94.17 ± 72.81 (13.0–120)
**Polyp size and number**	
Size of the largest polyp (cm)	1.20/1.53 ± 1.45 (0–18.0)
Number, n (%)	2.00/2.02 ± 0.881 (1–3)
N = 0	22 (4.1)
N = 1	199 (37.4)
N = 2	140 (26.3)
N = 3	171 (32.1)
**Polyp pathology**, n (%)	
No polyp	16 (3.0)
Inflammation, muscle prolapse, or juvenile polyp	17 (3.2)
Hyperplastic polyp	35 (6.6)
Sessile serrated adenoma	26 (4.9)
Tubular adenoma	291 (54.7)
Tubulovillous adenoma	67 (12.6)
Villous adenoma	18 (3.4)
Adenoma with high-grade dysplasia	33 (6.2)
Adenocarcinoma	29 (5.5)
**Polyp location** (largest one), n (%)	
Right colon (ascending and transverse colons and cecum)	205 (38.5)
Left colon (rectum, sigmoid, and descending colon), n (%)	327 (61.5)
**NAMPT-associated SNP genotype, n (%)**	
NAMPT-rs2302559 (CC/CT/TT)	451 (84.7)/78 (14.6)/3 (0.6)
NAMPT-rs61330082 (CC/CT/TT)	121(22.7)/293 (55.1)/118 (22.2)
NAMPT-rs10953502 (CC/CT/TT)	3 (0.6)/101 (19.0)/428 (80.8)
NAMPT-rs2058539 (CC/CA/AA)	3 (0.6)/100 (18.8)/429 (80.6)

BMI: body mass index; HOMA-IR: homeostasis model assessment-estimated insulin resistance; TC: total cholesterol; TGs: triglycerides; HDL-C: high-density lipoprotein cholesterol; UA: uric acid; NLR: neutrophil-to-lymphocyte ratio; HS-CRP: high-sensitivity C-reactive protein; CEA: carcinoembryonic antigen; eNAMPT: extracellular nicotinamide phosphoribosyltransferase; ALT: alanine aminotransferase; eGFR: estimated glomerular filtration rate; n: number of patients; N: number of polyps; SNP: single-nucleotide polymorphism.

**Table 2:** Univariate and multivariate analyses of factors associated with baseline eNAMPT levels.

	eNAMPT (ng/mL)			
	Univariate Analysis		Multivariate Analysis	
Variants	95% CI of Estimated β (Estimated β)	*p* Values	95% CI of Estimated β (Estimated β)	*p* Values
Sex (Male)	−0.835–0.674 (−0.147)	0.674		
Age (yr)	−0.016–0.041 (0.013)	0.377		
BMI	−0.046–0.145 (0.049)	0.308		
HOMA-IR	−0.384–0.451 (0.033)	0.874		
TC (mg/dL)	0.002–0.02 (0.011)	0.017 *	0.002–0.021 (0.011)	0.019 *
TGs (mg/dL)	−0.24–0.032(−0.001)	0.626		
HDL (mg/dL)	−0.024–0.032 (0.004)	0.781		
TG/HDL-C	−0.175–0.130 (−0.036)	0.613		
UA (mg/dL)	−0.034–0.389 (0.177)	0.1		
NLR	0.076–0.568 (0.322)	0.01 *	0.041–0.531 (0.286)	0.023 *
HS-CRP (mg/dL)	−0.09–0.077 (0.034)	0.123		
CEA (ng/mL)	−0.093–0.058 (−0.017)	0.648		
Platelet count (10^3^/µL)	−0.003–0.008 (0.002)	0.435		
ALT (U/L)	−0.16–0.011 (−0.002)	0.726		
eGFR (mL/min/1.73 m^2^)	−0.006–0.003 (−0.002)	0.524		
Polyp size (cm)	−0.059–0.383 (0.162)	0.149		
Polyp number	−0.035–0.681 (0.323)	0.076		
Polyp pathology	0.35–0.726 (0.538)	<0.001 *	0.355–0.788 (0.571)	<0.001 *
Polyp location	−0.306–0.731 (0.212)	0.42		
NAMPT-rs2302559 (CC:0/CT:1/TT:2)	−1.111–0.398 (−0.356)	0.354		
NAMPT-rs61330082 (CC:0/CT:1/TT:2)	−0.457–0.507 (0.025)	0.918		
NAMPT-rs10953502 (CC:0/CT:1/TT:2)	−0.542–0.984 (0.221)	0.57		
NAMPT-rs2058539 (CC:0/CA:1/AA:2)	−0.583–0.931 (0.174)	0.652		

eNAMPT: extracellular nicotinamide phosphoribosyltransferase; CI: confidence interval; BMI: body mass index, HOMA-IR: homeostasis model assessment-estimated insulin resistance; TC: total cholesterol; TGs: triglycerides; HDL-C: high-density lipoprotein cholesterol; UA: uric acid; NLR: neutrophil-to-lymphocyte ratio; HS-CRP: high-sensitivity C-reactive protein; CEA: carcinoembryonic antigen; ALT: alanine aminotransferase, eGFR: estimated glomerular filtration rate. * *p* < 0.05.

## Data Availability

The datasets generated and/or analyzed during the current study are available from the corresponding author on reasonable request.

## Supplementary Materials

The following supporting information can be downloaded at: https://www.mdpi.com/article/10.3390/cancers15061702/s1, Table S1. Primer sequences used in the single-nucleotide polymorphisms of NAMPT-rs61330082. Table S2. The 4 single-nucleotide polymorphisms evaluated in the study. Table S3. Univariate and multivariate analyses of factors associated with pathology of colonic polyp. Table S4. Univariate and multivariate analyses of factors associated with pathology of total cholesterol. Table S5. Univariate and multivariate analyses of factors associated with NLR. Figure S1. The linkage disequilibrium (LD) block structure consisted of 4 SNPs (rs61330082, rs2302559, rs10953502 and rs23058539) and showed a haplotype block constructed by 3 SNPs (rs2302559, rs10953502 and rs23058539).

## Abbreviations

ALT: alanine aminotransferase; BMI: body mass index; CEA: carcinoembryonic antigen; CRC: colorectal cancer; eGFR: estimated glomerular filtration rate; eNAMPT: extracellular nicotinamide phosphoribosyltransferase; HDL-C: high-density lipoprotein cholesterol; HOMA-IR: homeostasis model assessment-estimated insulin resistance; HS-CRP: high-sensitivity C-reactive protein; iNAMPT: intracellular NAMPT; NLR: neutrophil-to-lymphocyte ratio; NAD: nicotinamide adenine dinucleotide; NAMPT: Nicotinamide phosphoribosyltransferase; PMP: prominent malignant potential; SNP: single-nucleotide polymorphism; TC: total cholesterol, TGs: triglycerides, UA: uric acid.

## References

[1] Ferlay J., Shin H.-R., Bray F., Forman D., Mathers C., Parkin D.M. (2010). Estimates of worldwide burden of cancer in 2008: GLOBOCAN 2008. Int. J. Cancer.

[2] Lieberman D.A., Rex D.K., Winawer S.J., Giardiello F.M., Johnson D.A., Levin T.R. (2012). Guidelines for Colonoscopy Surveillance After Screening and Polypectomy: A Consensus Update by the US Multi-Society Task Force on Colorectal Cancer. Gastroenterology.

[3] Brenner H., Hoffmeister M., Stegmaier C., Brenner G., Altenhofen L., Haug U. (2007). Risk of progression of advanced adenomas to colorectal cancer by age and sex: Estimates based on 840 149 screening colonoscopies. Gut.

[4] Bond J.H. (2000). Polyp guideline: Diagnosis, treatment, and surveillance for patients with colorectal polyps. Practice Parameters Committee of the American College of Gastroenterology. Am. J. Gastroenterol..

[5] Rex D.K., Cutler C.S., Lemmel G.T., Rahmani E.Y., Clark D.W., Helper D.J., Lehman G.A., Mark D.G. (1997). Colonoscopic miss rates of adenomas determined by back-to-back colonoscopies. Gastroenterology.

[6] Robertson D.J., Lieberman D.A., Winawer S.J., Ahnen D.J., Baron J.A., Schatzkin A., Cross A.J., Zauber A.G., Church T.R., Lance P. (2013). Colorectal cancers soon after colonoscopy: A pooled multicohort analysis. Gut.

[7] Waye J.D., Bashkoff E. (1991). Total colonoscopy: Is it always possible?. Gastrointest. Endosc..

[8] Shah R., Jones E., Vidart V., Kuppen P.J.K., Conti J.A., Francis N.K. (2014). Biomarkers for Early Detection of Colorectal Cancer and Polyps: Systematic Review. Cancer Epidemiol. Biomark. Prev..

[9] Chen T.-H., Hsu C.-M., Hsu H.-C., Chiu C.-T., Su M.-Y., Chu Y.-Y., Chang M.-L. (2018). Plasminogen activator inhibitor-1 is associated with the metabolism and development of advanced colonic polyps. Transl. Res..

[10] Garten A., Schuster S., Penke M., Gorski T., de Giorgis T., Kiess W. (2015). Physiological and pathophysiological roles of NAMPT and NAD metabolism. Nat. Rev. Endocrinol..

[11] Imai S. (2010). “Clocks” in the NAD World: NAD as a metabolic oscillator for the regulation of metabolism and aging. Biochim. Biophys Acta..

[12] Gallí M., Van Gool F., Rongvaux A., Andris F., Leo O. (2010). The Nicotinamide Phosphoribosyltransferase: A Molecular Link between Metabolism, Inflammation, and Cancer. Cancer Res..

[13] Ho C., van der Veer E., Akawi O., Pickering J.G. (2009). SIRT1 markedly extends replicative lifespan if the NAD^+^salvage pathway is enhanced. FEBS Lett..

[14] van der Veer E., Ho C., O’Neil C., Barbosa N., Scott R., Cregan S.P., Pickering J.G. (2007). Extension of human cell lifespan by nicotinamide phosphoribosyltransferase. J. Biol. Chem..

[15] Romacho T., Azcutia V., Vázquez-Bella M., Matesanz N., Cercas E., Nevado J., Carraro R., Rodríguez-Mañas L., Sánchez-Ferrer C.F., Peiró C. (2009). Extracellular PBEF/NAMPT/visfatin activates pro-inflammatory signalling in human vascular smooth muscle cells through nicotinamide phosphoribosyltransferase activity. Diabetologia.

[16] Kitani T., Okuno S., Fujisawa H. (2003). Growth phase-dependent changes in the subcellular localization of pre-B-cell colony-enhancing factor^1^. FEBS Lett..

[17] Revollo J.R., Körner A., Mills K.F., Satoh A., Wang T., Garten A., Dasgupta B., Sasaki Y., Wolberger C., Townsend R.R. (2007). Nampt/PBEF/visfatin regulates insulin secretion in β cells as a systemic NAD biosynthetic enzyme. Cell Metab..

[18] Sun G., Bishop J., Khalili S., Vasdev S., Gill V., Pace D., Fitzpatrick D., Randell E., Xie Y.-G., Zhang H. (2007). Serum visfatin concentrations are positively correlated with serum triacylglycerols and down-regulated by overfeeding in healthy young men. Am. J. Clin. Nutr..

[19] Rahbar A., Nabipour I. (2014). The Relationship Between Dietary Lipids and Serum Visfatin and Adiponectin Levels in Postmenopausal Women. Endocrine, Metab. Immune Disord.—Drug Targets.

[20] Tsouma I., Kouskouni E., Demeridou S., Boutsikou M., Hassiakos D., Chasiakou A., Hassiakou S., Baka S. (2014). Correlation of visfatin levels and lipoprotein lipid profiles in women with polycystic ovary syndrome undergoing ovarian stimulation. Gynecol. Endocrinol..

[21] Chen L., Liu W., Lai S., Li Y., Wang X., Zhang H. (2013). Insulin resistance, serum visfatin, and adiponectin levels are associated with metabolic disorders in chronic hepatitis C virus-infected patients. Eur. J. Gastroenterol. Hepatol..

[22] Chang Y.C., Chang T.J., Lee W.J., Chuang L.-M. (2010). The relationship of visfatin/pre-B-cell colony-enhancing factor/nicotinamide phosphoribosyltransferase in adipose tissue with inflammation, insulin resistance, and plasma lipids. Metabolism.

[23] Grolla A.A., Travelli C., Genazzani A.A., Sethi J.K. (2016). Extracellular nicotinamide phosphoribosyltransferase, a new cancer metabokine. Br. J. Pharmacol..

[24] Ocvirk S., O’Keefe S.J. (2021). Dietary fat, bile acid metabolism and colorectal cancer. Semin. Cancer Biol..

[25] Chattopadhyay I., Gundamaraju R., Jha N.K., Gupta P.K., Dey A., Mandal C.C., Ford B.M. (2022). Interplay between Dysbiosis of Gut Microbiome, Lipid Metabolism, and Tumorigenesis: Can Gut Dysbiosis Stand as a Prognostic Marker in Cancer?. Dis. Markers.

[26] Motilva V., García-Mauriño S., Talero E., Illanes M. (2011). New paradigms in chronic intestinal inflammation and colon cancer: Role of melatonin. J. Pineal Res..

[27] Yang J., Zhang K., Song H., Wu M., Li J., Yong Z., Jiang S., Kuang X., Zhang T. (2016). Visfatin is involved in promotion of colorectal carcinoma malignancy through an inducing EMT mechanism. Oncotarget.

[28] Li H., Bai E., Zhang Y., Jia Z., He S., Fu J. (2017). Role of Nampt and Visceral Adiposity in Esophagogastric Junction Adenocarcinoma. J. Immunol. Res..

[29] Grolla A.A., Torretta S., Gnemmi I., Amoruso A., Orsomando G., Gatti M., Caldarelli A., Lim D., Penengo L., Brunelleschi S. (2015). Nicotinamide phosphoribosyltransferase (NAMPT/PBEF/visfatin) is a tumoural cytokine released from melanoma. Pigment. Cell Melanoma Res..

[30] Audrito V., Serra S., Brusa D., Mazzola F., Arruga F., Vaisitti T., Coscia M., Maffei R., Rossi D., Wang T. (2015). Extracellular nicotinamide phosphoribosyltransferase (NAMPT) promotes M2 macrophage polarization in chronic lymphocytic leukemia. Blood.

[31] Rosti V., Campanelli R., Massa M., Viarengo G., Villani L., Poletto V., Bonetti E., Catarsi P., Magrini U., Grolla A.A. (2016). Increased plasma nicotinamide phosphoribosyltransferase is associated with a hyperproliferative phenotype and restrains disease progression in MPN-associated myelofibrosis. Am. J. Hematol..

[32] Vora M., Ansari J., Shanti R.M., Veillon D., Cotelingam J., Coppola D., Shackelford R.E. (2016). Increased Nicotinamide Phosphoribosyltransferase in Rhabdomyosarcomas and Leiomyosarcomas Compared to Skeletal and Smooth Muscle Tissue. Anticancer. Res..

[33] Kumar A., Shenoy V., Buckley M.C., Durbin L., Mackey J., Mone A., Swaminath A. (2022). Endoscopic Disease Activity and Biologic Therapy Are Independent Predictors of Suboptimal Bowel Preparation in Patients with Inflammatory Bowel Disease Undergoing Colonoscopy. Dig. Dis. Sci..

[34] Hung S.-Y., Chen H.-C., Chen W.T.-L. (2020). A Randomized Trial Comparing the Bowel Cleansing Efficacy of Sodium Picosulfate/Magnesium Citrate and Polyethylene Glycol/Bisacodyl (The Bowklean Study). Sci. Rep..

[35] Ooi D.S.Q., Ong S.G., Heng C.K., Loke K.Y., Lee Y.S. (2016). In-vitro function of upstream visfatin polymorphisms that are associated with adverse cardiometabolic parameters in obese children. BMC Genom..

[36] Stastny J., Bienertova-Vasku J., Tomandl J., Tomandlova M., Zlamal F., Forejt M., Splichal Z., Vasku A. (2013). Association of genetic variability in selected regions in visfatin (NAMPT) gene with anthropometric parameters and dietary composition in obese and non-obese Central-European population. Diabetes Metab. Syndr. Clin. Res. Rev..

[37] Jian W.-X., Luo T.-H., Gu Y.-Y., Zhang H.-L., Zheng S., Dai M., Han J.-F., Zhao Y., Li G., Luo M. (2006). The visfatin gene is associated with glucose and lipid metabolism in a Chinese population. Diabet. Med..

[38] Zhang K., Zhou B., Zhang P., Zhang Z., Chen P., Pu Y., Song Y., Zhang L. (2013). Genetic variants in NAMPT predict bladder cancer risk and prognosis in individuals from southwest Chinese Han group. Tumor Biol..

[39] Benjamini Y., Hochberg Y. (1995). Controlling the False Discovery Rate: A Practical and Powerful Approach to Multiple Testing. J. R. Stat. Soc. Ser. B Methodol..

[40] Guo S.W., Thompson E.A. (1992). Performing the Exact Test of Hardy-Weinberg Proportion for Multiple Alleles. Biometrics.

[41] Chang M.-L., Lin Y.-S., Hsu C.-L., Chien R.-N., Fann C.S. (2021). Accelerated cardiovascular risk after viral clearance in hepatitis C patients with the NAMPT-rs61330082 TT genotype: An 8-year prospective cohort study. Virulence.

[42] Sung J.J.Y., Lau J.Y.W., Young G.P., Sano Y., Chiu H.-M., Byeon J.-S., Yeoh K.-G., Goh K.-L., Sollano J., Rerknimitr R. (2008). Asia Pacific consensus recommendations for colorectal cancer screening. Gut.

[43] Laird-Fick H.S., Chahal G., Olomu A., Gardiner J., Richard J., Dimitrov N. (2016). Colonic polyp histopathology and location in a community-based sample of older adults. BMC Gastroenterol..

[44] Pommergaard H.-C., Burcharth J., Rosenberg J., Raskov H. (2016). The association between location, age and advanced colorectal adenoma characteristics: A propensity-matched analysis. Scand. J. Gastroenterol..

[45] Kim E.C., Lance P. (1997). Colorectal polyps and their relationship to cancer. Gastroenterol. Clin. N. Am..

[46] Mouchemore K.A., Anderson R.L., Hamilton J.A. (2017). Neutrophils, G-CSF and their contribution to breast cancer metastasis. FEBS J..

[47] Fazeli M.S., Dashti H., Akbarzadeh S., Assadi M., Aminian A., Keramati M.R., Nabipour I. (2013). Circulating levels of novel adipocytokines in patients with colorectal cancer. Cytokine.

[48] Chung S.M., Kim K.O., Cho I.H., Kim T.N. (2017). Clinicopathological analysis and risk factors of advanced colorectal neoplasms incidentally detected by 18F-FDG PET-CT. Eur. J. Gastroenterol. Hepatol..

[49] Kim J.Y., Jung Y.S., Park J.H., Kim H.J., Cho Y.K., Sohn C.I., Jeon W.K., Kim B.I., Choi K.Y., Park N.I. (2016). Different risk factors for advanced colorectal neoplasm in young adults. World J. Gastroenterol..

[50] Cappell M.S. (1994). Safety and clinical efficacy of flexible sigmoidoscopy and colonoscopy for gastrointestinal bleeding after myocardial infarction. A six-year study of 18 consecutive lower endoscopies at two university teaching hospitals. Dig. Dis. Sci..

[51] Chen J., Sysol J.R., Singla S., Zhao S., Yamamura A., Valdez-Jasso D., Abbasi T., Shioura K.M., Sahni S., Reddy V. (2017). Nicotinamide Phosphoribosyltransferase Promotes Pulmonary Vascular Remodeling and Is a Therapeutic Target in Pulmonary Arterial Hypertension. Circulation.

[52] Jacobson-Brown P., Neuman M.G. (2004). Colorectal polyposis and immune-based therapies. Can. J. Gastroenterol..

[53] Kong M.Y., Li L.Y., Lou Y.M., Chi H.Y., Wu J.J. (2020). Chinese herbal medicines for prevention and treatment of colorectal cancer: From molecular mechanisms to potential clinical applica-tions. J. Integr. Med..

[54] Zhu L.Q., Zhang L., Zhang J., Chang G.L., Liu G., Yu D.D., Yu X.M., Zhao M.S., Ye B. (2021). Evodiamine inhibits high-fat diet-induced coli-tis-associated cancer in mice through regulating the gut microbiota. J. Integr. Med..

[55] Zhou H., Wang Y., Zhang Z., Xiong L., Liu Z., Wen Y. (2023). A novel prognostic gene set for colon adenocarcinoma relative to the tumor microenvironment, chemotherapy, and immune therapy. Front. Genet..

[56] Zweig M.H., Campbell G. (1993). Receiver-operating characteristic (ROC) plots: A fundamental evaluation tool in clinical medicine. Clin. Chem..

[57] Westreich D. (2012). Berkson’s bias, selection bias, and missing data. Epidemiology.

