# Supplementary material for: Extracellular Nicotinamide Phosphoribosyltransferase as a Surrogate Marker of Prominent Malignant Potential in Colonic Polyps: A 2-Year Prospective Study
Source: Cancers (Basel). 2023 Mar 10;15(6):1702. doi: 10.3390/cancers15061702 (PMC10046025; doi:10.3390/cancers15061702)
Supplement: Supplementary file 1 [file cancers-15-01702-s001.zip › cancers-2177890-supplementary.pdf]

Table S1. Primer sequences used in the single-nucleotide polymorphisms of NAMPT-rs61330082**.**

Genes	Primers	Sequences (5'->3')
NAMPT	NAMPT-F	5′ - TGTTTCAAACCTCGTT GCTG -3′
	NAMPT-R	5′ - GAGGCATGGCTGAGACTTCTA -3′

ScrFI for rs61330082—Allele C is cuttable, yielding two fragments of 65 and 138 bp; allele T is uncuttable, and the fragment is still 203 bp.

Table S2. The 4 single-nucleotide polymorphisms evaluated in the study.

Gene	SNP ID	ID number of TaqMan assays	Chromosome	Location	Risk allele	MAF	*P* HW[#]	*p* 1 *	*p* 2 *	*p* 3 *	Location in relation to NAMPT
NAMPT	rs61330082	NA	7q22.3	106248285	T	0.493	0.3639	0.918	0.478	0.217	promoter
NAMPT	rs10953502	C__31761492_10	7q22.3	106251985	C	0.102	0.7922	0.57	0.79	0.451	intron 10
NAMPT	rs2302559	C___2673293_10	7q22.3	106263458	T	0.077	0.9622	0.354	0.803	0.374	exon 7
NAMPT	rs2058539	C__11613113_10	7q22.3	106276191	C	0.099	0.8927	0.652	0.652	0.42	exon 11

NAMP: Nicotinamide phosphoribosyltransferase; NA: not accessible; MAF: minor allele frequency; # *P* value of the Chi-square test on the Hardy-Weinberg equilibrium; *The genetic model is an additive model (value1: for baseline eNAMPT levels; value 2: for total cholesterol levels; value 3: for homeostasis model assessment-estimated insulin resistance).

Table S3. Univariate and multivariate analyses of factors associated with pathology of colonic polyp.

	Pathology of colonic polyp			
	Univariate analysis		Multivariate analysis	
Variants	95% CI of estimated β (estimated β)	*p* values	95% CI of estimated β (estimated β)	*p* values
Gender (Male)	-0.385-0.379 (-0.003)	0.986		
Age (yr)	0.02-0.051 (0.035)	<0.001*	0.013-0.041 (0.027)	<0.001*
BMI	-0.052-0.076 (0.012)	0.716		
HOMA-IR	-0.14-0.301 (0.08)	0.473		
TC (mg/dL)	-0.004-0.006 (0.001)	0.715		
TGs (mg/dL)	-0.003-0.001(-0.001)	0.463		
HDL-C (mg/dL)	-0.007-0.023 (0.008)	0.303		
TG/HDL-C	-0.1-0.053 (-0.024)	0.544		
eNAMPT (ng/mL)	0.107-0.223 (0.165)	<0.001*	0.091-0.186 (0.139)	<0.001*
NLR	-0.0182-0.183 (0.051)	0.453		
HS-CRP (mg/dL)	-0.009-0.037 (0.014)	0.233		
CEA (ng/mL)	-0.039-0.046 (0.003)	0.873		
Platelet count (10^3/μL)	-0.004-0.002 (-0.001)	0.424		
ALT (U/L)	-0.009-0.006 (-0.002)	0.674		
eGFR (mL/min/1.73 m^2)	-0.003-0.002 (0.000)	0.856		
Polyp size (cm)	0.246-0.446 (0.346)	<0.001*	0.195-0.386 (0.291)	<0.001*
Polyp number	-0.002-0.349 (0.174)	0.052		
Polyp location	-0.052-0.475 (0.211)	0.116		
NAMPT-rs2302559 (CC:0/CT:1/TT:2)	-0.708 -0.331 (-0.186)	0.475		
NAMPT-rs61330082 (CC:0/CT:1/TT:2)	-0.185-0.35 (0.083)	0.543		
NAMPT-rs10953502 (CC:0/CT:1/TT:2)	-0.238 -0.624 (0.193)	0.379		
NAMPT-rs2058539 (CC:0/CA:1/AA:2)	-0.202-0.663 (0.231)	0.294		

eNAMPT: extracellular nicotinamide phosphoribosyltransferase; CI: confidence interval; BMI: body mass index, HOMA-IR: homeostasis model assessment-estimated insulin resistance; TC: total cholesterol; TG: triglycerides; HDL-C: high-density lipoprotein-cholesterol; UA: uric acid; NLR: neutrophil-to-lymphocyte ratio; HS-CRP: high sensitivity C-reactive protein; CEA: carcinoembryonic antigen; ALT: alanine aminotransferase, eGFR: estimated glomerular filtration rate. *, $p<0.05$.

Supplementary Table S4. Univariate and multivariate analyses of factors associated with pathology of total cholesterol.

	Total cholesterol (mg/dL)			
	Univariate analysis		Multivariate analysis	
Variants	95% CI of estimated β (estimated β)	*p* values	95% CI of estimated β (estimated β)	*p* values
Gender (Male)	-0.879-8.45 (-0.168 3)	0.97		
Age (yr)	-0.634-0.093 (-0.27)	0.144		
BMI	-1.141-1.585 (0.222)	0.749		
HOMA-IR	-3.71-0.501 (0.653)	0.768		
UA (mg/dL)	-3.79-1.32 (-1.23)	0.342		
TGs (mg/dL)	-0.001-0.099 (0.049)	0.057		
HDL-C (mg/dL)	0.365-1.007 (0.681)	<0.001*	0.332-1.006 (0.669)	<0.001*
TG/HDL-C	-1.41-1.943 (0.269)	0.752		
eNAMPT (ng/mL)	0.294-2.914 (1.604)	0.017*	0.158-2.74 (1.447)	0.028*
NLR	-2.8-3.174 (0.183)	0.904		
HS-CRP (mg/dL)	-0.935 -0.017 (-0.432)	0.092		
CEA (ng/mL)	-0.845-1.157 (0.156)	0.759		
Platelet count (10^3/μL)	0.027-0.157 (0.092)	0.006*	0.035-0.167 (0.101)	0.003*
ALT (U/L)	-0.04-0.28 (0.12)	0.14		
eGFR (mL/min/1.73 m^2)	-0.046-0.065 (0.009)	0.738		
Polyp size (cm)	-0.548 --0.152 (-2.818)	0.038*	-5.63--0.469 (-3.048)	0.021*
Polyp number	1.27-10.025 (5.647)	0.012*	1.864-10.727 (6.295)	0.006*
Polyp location	-0.3.197 -9.446 (3.124)	0.332		
NAMPT-rs2302559 (CC:0/CT:1/TT:2)	-9.55 -12.32 (1.385)	0.803		
NAMPT-rs61330082 (CC:0/CT:1/TT:2)	-3.72-7.92 (2.1)	0.478		
NAMPT-rs10953502 (CC:0/CT:1/TT:2)	-10.51-8.00 (-1.254)	0.79		
NAMPT-rs2058539 (CC:0/CA:1/AA:2)	-11.56-7.12 (-2.07)	0.658		

eNAMPT: extracellular nicotinamide phosphoribosyltransferase; CI: confidence interval; BMI: body mass index, HOMA-IR: homeostasis model assessment-estimated insulin resistance; TC: total cholesterol; TG: triglycerides; HDL-C: high-density lipoprotein-cholesterol; UA: uric acid; NLR: neutrophil-to-lymphocyte ratio; HS-CRP: high sensitivity C-reactive protein; CEA: carcinoembryonic antigen; ALT: alanine aminotransferase, eGFR: estimated glomerular filtration rate. *, $p<0.05$; TG/HDL-C and NAMPT-rs2302559 had been eliminated from multivariate analyses due to high variance inflation faction values.

Table S5. Univariate and multivariate analyses of factors associated with NLR.

	NLR			
	Univariate analysis		Multivariate analysis	
Variants	95% CI of estimated β (estimated β)	*p* values	95% CI of estimated β (estimated β)	*p* values
Gender (Male)	-0.88-0.589 (-0.145)	0.686		
Age (yr)	-0.028-0.026 (-0.001)	0.939		
BMI	-0.088-0.136 (0.024)	0.664		
HOMA-IR	-0.402-0.278 (-0.062)	0.702		
TC (mg/dL)	-0.001-0.003 (-0.003)	0.314		
TGs (mg/dL)	-0.003-0.004(0.000)	0.866		
HDL-C (mg/dL)	-0.001-0.046 (0.018)	0.193		
TG/HDL-C	-0.099-0.122 (0.011)	0.833		
eNAMPT (ng/mL)	0.016 -0.12 (0.068)	0.01*	0.03-0.183 (0.107)	0.007*
UA (mg/dL)	0.026-0.228 (0.127)	0.014*	-0.089-0.205 (0.058)	0.436
HS-CRP (mg/dL)	0.031-0.097 (0.064)	<0.001*	0.014-0.016 (0.065)	0.013*
CEA (ng/mL)	0.005-0.091 (0.048)	0.029*	-0.004-0.085 (0.04)	0.076
Platelet count (10^3/μL)	-0.001-0.004 (0.002)	0.139		
ALT (U/L)	-0.007-0.006 (0.000)	0.924		
eGFR (mL/min/1.73 m^2)	-0.002-0.002 (0.000)	0.863		

Polyp size (cm)	-0.071-0.141 (0.035)	0.517
Polyp number	-0.072-0.285 (0.107)	0.241
Polyp location	-0.332-0.175 (-0.078)	0.544
NAMPT-rs2302559 (CC:0/CT:1/TT:2)	-0.607 -0.313 (-0.147)	0.53
NAMPT-rs61330082 (CC:0/CT:1/TT:2)	-0.209 -0.276 (0.034)	0.784
NAMPT-rs10953502 (CC:0/CT:1/TT:2)	-0.161 -0.584 (0.212)	0.264
NAMPT-rs2058539 (CC:0/CA:1/AA:2)	-0.371-0.915 (0.272)	0.25

eNAMPT: extracellular nicotinamide phosphoribosyltransferase; CI: confidence interval; BMI: body mass index, HOMA-IR: homeostasis model assessment-estimated insulin resistance; TC: total cholesterol; TG: triglycerides; HDL-C: high-density lipoprotein-cholesterol; UA: uric acid; NLR: neutrophil-to-lymphocyte ratio; HS-CRP: high sensitivity C-reactive protein; CEA: carcinoembryonic antigen; ALT: alanine aminotransferase, eGFR: estimated glomerular filtration rate. *, $p<0.05$.

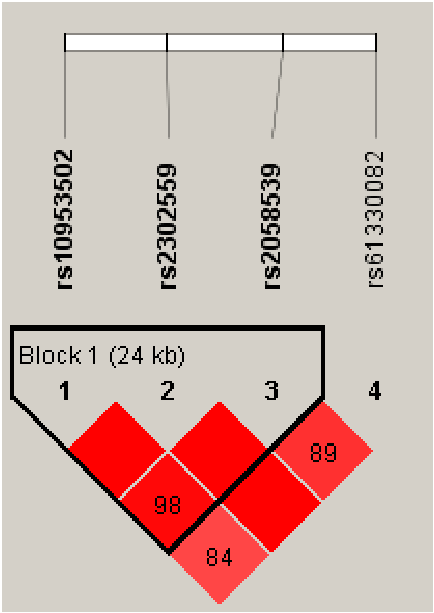

Supplementary Figure S1. The linkage disequilibrium (LD) block structure consisted of 4 SNPs (rs61330082, rs2302559, rs10953502 and rs23058539) and showed a haplotype block constructed by 3 SNPs (rs2302559, rs10953502 and rs23058539).
